# VMN growth hormone-releasing hormone receptor regulation of counterregulatory transmission

**DOI:** 10.20935/acadbiol7623

**Published:** 2025

**Authors:** Subash Sapkota, Rami Shrestha, Sushma Katakam, Sagor C. Roy, Karen P. Briski

**Affiliations:** 1 School of Basic Pharmaceutical and Toxicological Sciences, College of Pharmacy, University of Louisiana Monroe, Rm 356 Bienville Building, 1800 Bienville Drive, Monroe, LA 71201, USA.

**Keywords:** growth hormone-releasing hormone receptor, ventromedial hypothalamic nucleus, insulin-induced hypoglycemia, single-cell multiplex qPCR, corticosterone, sex differences

## Abstract

Ventromedial hypothalamic nucleus (VMN) growth hormone-releasing hormone (Ghrh) neurotransmission governs counterregulatory hormone release. Recent studies document Ghrh control of hypoglycemia-sensitive counterregulatory neurotransmitter expression in dorsomedial VMN (VMNdm) Ghrh/steroidogenic factor-1 (SF-1) neurons. In this study, Ghrh receptor (Ghrh-R) gene silencing was implemented in vivo to determine if VMN Ghrh-R shapes counterregulation. Intra-VMN Ghrh-R siRNA augmented corticosterone secretion in vehicle or insulin-injected male rats, but this hormone was correspondingly refractory or inhibited in eu- versus hypoglycemic females. In each sex, gene knockdown up- or down-regulated baseline glucagon and growth hormone (GH) release, but hypoglycemia reversed the direction of Ghrh-R control of each hormone. Single-cell laser catapult-microdissected VMNdm Ghrh/SF-1 neuron multiplex qPCR analysis revealed contrary VMN Ghrh-R gene-silencing effects on eu- versus hypoglycemic SF-1 mRNA levels. In both sexes, Ghrh-R siRNA up-regulated mRNAs encoding counterregulation-repressive (γ-aminobutyric acid) or -enhancing (nitric oxide) transmitter protein markers, unrelated to plasma glycemic profiles. Ghrh-R regulation of Ghrh gene transcription was absent (euglycemic) or stimulatory (hypoglycemic) in females, and receptor control of glutaminase mRNA, a marker for the counterregulatory-augmenting neurochemical glutamate, was lost in hypoglycemic males. Ghrh-R gene silencing caused uniform up-regulation of 5′-AMP-activated protein kinase alpha-2 (AMPKα2) mRNA in each sex, independent of glucose status, but caused dissimilar changes in AMPKα1 transcription in eu- versus hypoglycemic females. The outcomes provide novel evidence that VMN Ghrh-R signaling imposes glucose-dependent control of counterregulatory hormone secretion and distinctive VMNdm neuron counterregulatory transmitter marker gene profiles. Data infer that this metabolic control may involve SF-1 (both sexes)- and AMPKα1 (female)-dependent mechanisms.

## 1. Introduction

Maintenance of glucose homeostasis requires integrated autonomic, neuroendocrine, and behavioral outflow to manage glucose uptake, utilization, synthesis, and storage; insulin and counterregulatory hormone secretion; and food intake [1–5]. The hypothalamus, the principal visceral motor command center in the brain, coordinates these vital effector functions [6–12]. The ventromedial hypothalamic nucleus (VMN), a prominent structural constituent of the mediobasal hypothalamus, is a critical metabolic sensory and integrative component of the brain glucostatic network [13–15]. There is considerable interest in characterizing the VMN neuron population(s) that function within this neural circuitry and identifying regulatory metabolic, endocrine, and neurochemical cues that shape their input. Dorsomedial VMN (VMNdm) growth hormone-releasing hormone (Ghrh)/steroidogenic factor-1 (SF-1/NR5A1) neurons express the counterregulation hormone-enhancing neuropeptide Ghrh alongside genes that encode enzyme markers for several neurotransmitters that augment or constrain counterregulatory hormone secretion, namely the labile gas nitric oxide (NO) and the amino acids glutamate (Glu) and γ-aminobutyric acid (GABA) [16]. Crucially, transcription of these co-expressed neurotransmitter gene profiles is subject to Ghrh neuromodulatory control. This consolidated regulation of multiple neurochemicals of diverse chemical structure, spatial, and temporal profiles supports the notion that Ghrh may shape VMNdm Ghrh neuron provision of complex, integrated multi-modal input to the brain glucose-regulatory network. Evidence that Ghrh regulation of distinctive counterregulatory transmitter marker gene profiles is reliant upon systemic glucose profiles, i.e., is gained or lost by induction of insulin (INS)-induced hypoglycemia (IIH), infers that this neuropeptide signal may coordinate VMNdm Ghrh neuron transmission output during glucose dyshomeostasis [16].

Neither the neuroanatomical site(s) wherein Ghrh imposes control of counterregulatory hormone output nor the identity(-ies) of local cellular target(s) for this neuropeptide action are known. A plausible theory is that Ghrh may act on VMN targets, namely on VMNdm Ghrh/SF-1 neurons to direct counterregulation, as these neurons express Ghrh receptor (Ghrh-R) mRNA. Recent research used combinative in situ immunocytochemistry/laser catapult microdissection/single-cell multiplex qPCR techniques, within the context of a validated in vivo experimental model for IIH, to address the hypothesis that VMN Ghrh-R-dependent mechanisms govern eu- and/or hypoglycemic patterns of counterregulatory hormone release. Adult rats were pretreated with bilateral administration of self-delivering Accell^™^ Ghrh-R or control/scramble siRNA to the VMN prior to subcutaneous (*sc*) vehicle or INS injection. To align with the current U.S. National Institutes of Health policy’s emphasis on the evaluation of sex as an important biological variable, the present study design incorporated adult rats of each sex to investigate the corollary notion that Ghrh-R may exert sex-dimorphic control of eu- and/or hypoglycemia-associated counterregulatory hormone profiles and VMNdm Ghrh neuron counterregulatory neurochemical marker gene expression. Ghrh neurons were collected by laser catapult microdissection from the VMNdm of each experimental subject across all treatment groups for single-cell multiplex qPCR analysis of Ghrh, neuronal nitric oxide synthase (nNOS), glutamate decarboxylase_67_ (GAD1/GAD_67_), glutamate decarboxylase_65_ (GAD2/GAD_65_), and glutaminase (GLS) transcript profiles. This approach allowed us to examine whether VMN Ghrh-R imposes glucose status-specific Ghrh control of distinctive counterregulatory transmitter marker mRNAs.

The transcription factor SF-1/NR5A1 is uniquely expressed in the VMN, where it shapes establishment of VMN nerve cell population phenotypes during development [17–20]. SF-1 gene repression negatively affects VMN cytoarchitectural organization, culminating in thermogenic, metabolic, and reproductive [21–26]. SF-1 expression within the boundaries of the VMN is not ubiquitous, as it occurs in the VMNdm and central VMN, but not ventrolateral VMN. Our recent work documents SF-1 regulation of VMNdm Ghrh/SF-1 counterregulatory neurotransmitter marker gene transcription. We were aptly motivated here to investigate here whether this transcription factor mRNA profile is controlled by Ghrh-R signaling under eu- and/or hypoglycemic conditions in one or both sexes.

The ultra-sensitive energy gauge 5′-AMP-activated protein kinase (AMPK) is activated by phosphorylation when the cellular AMP/ATP ratio increases [27–30]. Hypothalamic AMPK supplies vital cues on cell energy stability to neural pathways that govern bodily energy balance [31]. Ventromedial hypothalamic AMPK is implicated in neural regulation of hypoglycemic patterns of counterregulatory hormone outflow [32, 33]. VMNdm Ghrh/SF-1 neurons may monitor intrinsic energy balance as these cells express AMPK protein. The AMPK alpha catalytic subunit exists as two isoforms, e.g., Prkaa1/AMPK-alpha1 (AMPKα1) and Prkaa2/AMPK-alpha2 (AMPKα2). These variants are activated to a comparable extent when AMP levels rise, but each exhibits distinctive, dissimilar substrate specificity, which likely results in dissimilar effects on cell function by phosphorylation of different target proteins [34]. Recent studies showed that Ghrh exerts a positive tone on AMPKα1 and AMPKα2 gene expression in male rats, irrespective of systemic glucose profiles [35]. In contrast, in the female, this neuropeptide signal has little effect on AMPKα1 transcription and correspondingly stimulates or inhibits AMPKα2 transcription in eu- versus hypoglycemic female rats. In this research, we investigated the hypothesis that Ghrh-R may regulate VMNdm Ghrh/SF-1 neuron AMPKα variant mRNAs according to systemic glucose status in one or both sexes.

## 2. Materials and methods

### Animals:

Adult Sprague Dawley rats were housed 2–3 per shoe-box cage, according to sex, under a 14 h light—10 h dark cycle (lights on at 05.00 h). Animals had free access to standard laboratory chow and tap water over the course of the study and were acclimated to handling by daily gentling before the onset of experimentation. Study protocols and procedures were conducted in accordance with the NIH Guide for Care and Use of Laboratory Animals, 8th Edition, with ULM Institutional Animal Care and Use Committee approval (Approval code: 21NOV-KPB-01. Date of approval 6 Nov 2021).

### Experimental Design:

On Study Day 1, animals of each sex were randomly assigned to four treatment groups, as described in Table 1 (n = 6 male and n = 6 female rats/treatment group). The rats were anesthetized by ketamine/xylazine (9.0 mg ketamine/1.0 mg xylazine/0.1 mL/100 g bw) injection prior to bilateral intraVMN injection (total volume: 1.0 μL; infusion rate: 3.6 μL/min; three-dimensional coordinates: −2.5 mm posterior to bregma, 0.6 mm lateral to midline, 9.0 mm ventral to skull surface) of scramble (SCR) siRNA (500 pmol; Accell Control Pool Non-Targeting; prod. no. D-001910–10-20; Horizon Discovery, Waterbeach, UK) or Ghrh-R siRNA (500 pmol; Accell rat Ghrh-R siRNA, set of 4; prod. no. EQ-089494–00-0010; Horizon Disc.), as described [16]. siRNA injections were performed using a 33-gauge Neuros syringe (prod. no. 53496; Stoelting Co., Wood Dale, IL, USA), guided by a Neurostar stereotactic Drill and Injection Robot (Neurostar, Tubingen, Germany). The current study design included standardization of circulating plasma estradiol concentrations in female subjects to minimize variability owing to disparate endogenous estradiol secretion at distinct estrous cycle stages. Here, anesthetized female rats were bilaterally ovariectomized (OVX) and implanted with a *sc* silastic capsule (0.062 in. *i.d*./ 0.125 in. *o.d*.; 10 mm/100 g bw) filled with 30 ug 17β estradiol-3-benzoate/mL safflower oil immediately after targeted intracranial siRNA administration. The mean plasma estradiol levels achieved by this replacement protocol (i.e., 22 pg/mL [36]) approximate concentrations measured during metestrus stage in 4-day cycling ovary-intact rats adult rat [37]. After surgery, rats were injected with ketophen (*sc*; Zoetis Inc., Kalamazoo, MI, USA) and enrofloxacin (intramuscular; Bayer HealthCare LLC, Animal Health Division, Shawnee Mission, KS, USA) and treated by topical application of 0.25% bupivacaine to closed incisions; animals were moved to single-occupancy cages after full recovery from anesthesia. On Study Day 7, male and female rats were injected *sc* at 09.00 h with vehicle (V; sterile diluent; Eli Lilly & Co., Indianapolis, IN, USA) or neutral protamine Hagedorn insulin (INS; 10.0 U/kg bw; Eli Lilly [38]), then sacrificed by rapid decapitation one hour post-injection. Individual brains were dissected whole, then snap-frozen by immersion in liquid nitrogen-cooled isopentane for storage at −80 °C.

### Laser Catapult Microdissection of VMNdm Ghrh Neurons:

Serial 10 micron (μm) thick fresh frozen transverse sections of the VMN were collected between −1.80 and −2.3 mm posterior to *bregma* and mounted on polyethylene naphthalate membrane-coated slides (prod. no. 415190–9041-000; Carl Zeiss Microscopy LLC, White Plains, NY, USA). Tissues were fixed with ice-cold acetone (5 min), blocked with 1.5% normal goat serum (prod. no. S-2000, Vector Laboratories, Burlingame, CA, USA) in Tris-buffered saline, pH 7.4, (TBS), 0.05% Triton X-100 (2 h), then incubated with a rabbit primary anti-preproGhrh antiserum (prod. No. PA5–102738, 1:2000; Invitrogen, Waltham, MA, USA) (48–72 h; 4 °C). Next, sections were incubated with a horseradish peroxidase-labeled goat anti-rabbit secondary antibody (prod. no. PI-1000, 1:1000; Vector Lab.; 1 h), then exposed to ImmPACT 3,3-diaminobenzidine peroxidase substrate kit reagents (prod. no. SK-4105; Vector Lab.). For each animal, individual Ghrh-immunoreactive (ir)-positive neurons were detached and propelled from tissue sections using a Zeiss P.A.L.M. UV-A microlaser IV system, as reported [39–41], and collected into separate adhesive caps (prod. no. 415190–9181-000; Carl Zeiss) containing lysis buffer (4 μL; Single Shot Cell Lysis Kit, prod. no. 1725080; Bio-Rad Laboratories, Hercules, CA, USA) for multiplex quantitative gene expression analysis. Additional Ghrh-ir neurons were collected into Western blot lysis buffer (2% sodium dodecyl sulfate [SDS], 0.05 M dithiothreitol, 10.0% glycerol, 1.0 mM EDTA, 60.0 mM Tris-HCl, pH 7.2), as described [39, 40] for Western blot confirmation of efficacy of Ghrh-R siRNA down-regulation of gene production expression

### Single-Cell Multiplex Quantitative Reverse Transcription PCR (RT-qPCR) Analysis:

#### Complementary DNA (cDNA) Synthesis and Amplification:

For each animal, n = 2 Ghrh-ir neurons were collected for single-cell gene expression analysis. Single-cell lysates were centrifuged (3000 rpm; 4 °C) prior to incubation in an iCyclerQ RT-PCR Detection System (Bio-Rad) at sequential 25 °C (10 min) and 75 °C (5 min) temperatures. Sample RNA integrity, quantity, and purity were determined by NanoDrop spectrophotometry (prod. no. ND-ONE-W, ThermoFisherScientific, Waltham, MA, USA). Single-cell mRNA samples were reverse-transcribed to cDNA by addition of 1.5 μL cDNA synthesis buffer (iScript^™^ Advanced cDNA Synthesis Kit. prod. No. 1725038; Bio-Rad) before they were incubated first at 46 °C (20 min), followed by 95 °C (1 min) [42]. A preamplification master mix was prepared by combining PrimePCR^™^ PreAmp for SYBR^®^ Green Assays for Ghrh-R (prod. no. qRnoCED0003825), Ghrh (prod. no. qRnoCID0007723), SF-1/NR5A1 (prod. no. qRnoCID0001458), GAD_67_/GAD1 (prod. no. qRnoCID0004554), GAD_65_/GAD2 (pro. no. qRnoCID0003485), nNOS/NOS1 (prod. no. qRnoCED0009301), GLS (prod. no. qRnoCID0007756), AMPKα1 (prod. no. qRnoCID0001262), AMPKα2 (prod. no. qRnoCID0006799), and the housekeeping gene GAPDH (prod. no. qRnoCID0057018; Bio-Rad) with SsoAdvanced^™^ PreAmp Supermix (prod. no. 1725160; Bio-Rad). Pre-amplified cDNA was produced by adding 9.5 μL preamplification master mix to individual cDNA samples prior to thermal cycler incubation at 95 °C (3 min), followed by 18 cycles of incubation at 95 °C (15 s), then 58 °C (4 min). Pre-amplified cDNA samples were diluted with IDTE (185 μL; prod. No. 11–05-01–05; 1X TE solution; Integrated DNA Technologies, Inc., Coralville, IA). *RT-qPCR Analysis*: PCR samples were prepared by combining Bio-Rad primers [Ghrh-R (0.5 μL; prod. no. qRnoCED0003825), Ghrh (0.5 μL; prod. no. qRnoCID0007723), SF-1/NR5A1 (0.5 μL; prod. no. qRnoCID0001458), GAD1/GAD_67_ (0.5 μL; prod. no. qRnoCID0004554), GAD2/GAD_65_ (0.5 μL; prod. no. qRnoCID0003485), GLS (0.5 μL; prod. no. qRnoCID0007756), nNOS/NOS1 (0.5 μL; prod. no. qRnoCED0009301), AMPKα1 (0.5 μL; prod. no. qRnoCID0001262), AMPKα2 (0.5 μL; prod. no. qRnoCID0006799), and GAPDH (0.5 μL; prod. no. qRnoCID0057018)], cDNA sample (2 μL), and iTaq^™^ Universal SYBR^®^ Green Supermix (5 μL, prod. no. 1725121; Bio-Rad). PCR samples were added to individual wells of hard-shell 384-well PCR plates (prod. no. HSP3805, Bio-Rad) for analysis in a CFX384^™^ Touch Real-Time PCR Detection System (Bio-Rad) under the following conditions: initial 30 s 95 °C denaturation, followed by 40 cycles of (1) 3 s incubation at 95 °C and (2) 30 s incubation at 60 °C for GAD_67_; 59.9 °C for GAD_65_; 59.1 °C for SF-1/NR5A1; 58.8 °C for GLS; 58.5 °C for Ghrh; 58 °C for nNOS/NOS1; or 57.3 °C for GAPDH, respectively. Melt curve analyses were performed to detect nonspecific products and primer dimers. Data were normalized using the comparative Ct (2^−ΔΔCt^) method [43].

### Western blot Analysis of Ghrh-R Protein in Laser-Microdissected Ghrh-ir Neurons for Confirmation of siRNA Gene Knockdown Efficacy:

For each treatment group, triplicate cell lysate pools (n = 50 Ghrh-ir neurons/pool/treatment group) were created for Ghrh-R protein analysis. Sample pool proteins were separated by electrophoresis in Bio-Rad TGX 12% stain-free gels (prod. no. 1610185, Bio-Rad Laboratories Inc., Hercules, CA, USA). Stain-Free imaging technology for total protein measurement was used as the loading control [40, 44]. After electrophoresis, gels were activated by UV light (1 min) in a Bio-Rad ChemiDoc MP Imaging System for quantification of individual in-lane total protein. Proteins were transferred to 0.45 μm PVDF-Plus membranes (prod. no. 121639; Data Support Co., Panorama City, CA, USA), for FreedomRocker^™^ Blotbot^®^ (Next Advance, Inc., Troy, NY, USA) automated wash and antibody incubation processing. Non-specific immunoreagent binding was minimized by membrane blocking with Tris-buffered saline, pH 7.4, 10 mM tris hydrochloride, 50 mM sodium chloride (TBS) supplemented with 0.1% Tween-20 and 2% bovine serum albumin. Membranes were sequentially incubated with a rabbit anti-Ghrh-R primary polyclonal antiserum (prod. no. PA5–121195; 1:1000; Invitrogen) (36–42 h; 4 °C), followed by goat anti-rabbit horseradish peroxidase-labeled secondary antibodies (1:5000; prod. no. NEF812001EA; PerkinElmer, Waltham, MA, USA) before exposure to SuperSignal West Femto chemiluminescent substrate (prod. no. 34096; ThermoFisherSci.), a proprietary trihalo compound that is directly incorporated into Bio-Rad Stain-Free gel chemistry’s inherent fluorescence but renders all in-gel proteins fluorescent and thus can be measured by its optical density (O.D.) upon UV photoactivation. After summation of individual protein O.D. values in a single lane, the chemiluminescence O.D. value obtained for the target protein band was normalized to summed total in-lane protein by ChemiDoc MP Image Lab^™^ 6.0.0 software. The figures presented here denote mean normalized O.D. measures at the Y axis. Each Western blot analysis employed precision plus protein molecular weight dual color standards (prod. no. 161–0374, Bio-Rad).

### Plasma Glucose and Counter-Regulatory Hormone Analyses:

Plasma glucose concentrations were measured in duplicate for each subject using an ACCU-CHECK Aviva-plus glucometer (Roche Diagnostic Corporation, Indianapolis, IN) [38]. Circulating corticosterone (prod. no. ADI-900–097; Enzo Life Sciences, Inc., Farmingdale, NY) and glucagon (prod. no. EZGLU-30K, EMD Millipore, Billerica, MA) levels were determined in duplicate using commercial ELISA kit reagents, as reported [44]. Serum GH levels were assayed in GH Rat ELISA Kit reagents (prod. no. KRC5311; Invitrogen/ThermoFisherSci., Waltham, MA, USA] [16].

### Statistics:

Mean normalized mRNA, normalized Ghrh-R protein, glucose, and counterregulatory hormone values were analyzed among treatment groups by three-way analysis of variance and Student–Newman–Keuls post hoc test. Differences of *p* < 0.05 were considered significant. In each figure, statistical differences between specific pairs of treatment groups are denoted as follows: * *p* < 0.05; ** *p* < 0.01; *** *p* < 0.001.

## 3. Results

The neuroanatomical site(s) and cellular target(s) for Ghrh control of counterregulatory hormone secretion are not known. In our research, we used in vivo Ghrh-R gene-silencing tools to address the premise that VMN Ghrh-R may impose glucose status-specific Ghrh management of plasma counterregulatory hormone profiles in one or both sexes. Ghrh regulation of distinctive VMNdm Ghrh/SF-1 neuron counterregulatory transmitter marker gene expression varies according to systemic glucose profiles, inferring that this neuropeptide may coordinate VMNdm Ghrh neuron neurochemical signal responses to glucose dyshomeostasis. Here, individual laser catapult-microdissected VMNdm Ghrh/SF-1 neurons were analyzed by single-cell multiplex qPCR to examine whether VMN Ghrh-R exerts differential control of counterregulatory transmitter marker mRNAs during eu- versus hypoglycemia in each sex.

**Figure 1 •:**
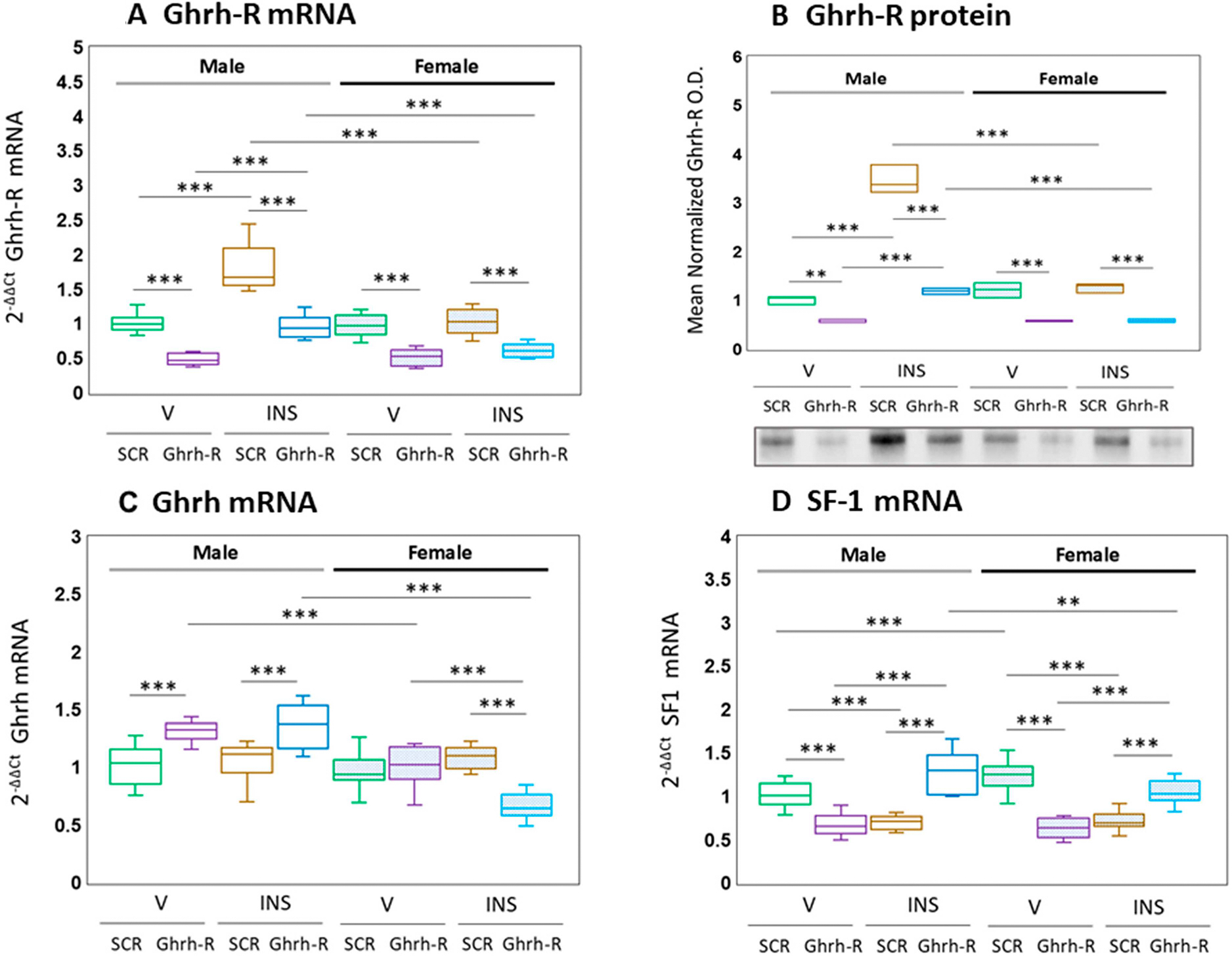
Effects of ventromedial hypothalamic nucleus (VMN) growth hormone-releasing hormone receptor (Ghrh-R) gene knockdown on Ghrh-R, Ghrh and steroidogenic factor-1 (SF-1) gene expression in male or female dorsomedial VMN (VMNdm) Ghrh-immunopositive neurons. Groups of male and female rats (n = 6 males and n = 6 females per group) were pretreated by bilateral intra-VMN scramble (SCR) or Ghrh-R siRNA administration seven days before subcutaneous (sc) injection of vehicle (V) or neutral protamine Hagedorn insulin (INS; 10.0 U/kg bw). Brain tissue was collected one hour post-injection. Individual Ghrh-immunopositive neurons were laser catapult-microdissected from 10-micron-thick fresh frozen sections cut through the VMNdm for multiplex single-cell qPCR analyses. mRNA data were normalized to the housekeeping gene GAPDH by the 2^ΔΔCt^ method [43]. Data are depicted here in box-and-whisker plot format, which displays the median, lower and upper quartiles, and lower and upper extremes of a data set. Plots depict normalized Ghrh-R (**A**), Ghrh (**C**), or SF-1 (**D**) mRNA measures + S.E.M. for male (*at left*) and female (*at right*) rat treatment groups. Treatment groups are identified as follows: SCR siRNA/V (green box-and-whisker plots, male: no fill, n = 12; female: stippled fill, n = 12); Ghrh-R siRNA/V (purple box-and-whisker plots; male: no fill, n = 12; female: stippled fill, n = 12); SCR siRNA/INS (brown box-and-whisker plots; male: no fill, n = 12; female: stippled fill, n = 12); Ghrh-R siRNA/INS (blue box-and-whisker plots; male: no fill, n = 12; female: stippled fill, n = 12). For each treatment group, aliquots of micropunched VMNdm tissue obtained from individual subjects were combined to create triplicate sample pools for Western blot analysis of Ghrh-R protein. The data in (**B**) depict SF-1 protein optical density values for the treatment groups described above. mRNA and protein data were analyzed by three-way ANOVA and a Student–Neuman–Keuls post hoc test. Statistical differences between discrete pairs of treatment groups are denoted as follows: ** *p* < 0.01; *** *p* < 0.001.

Figure 1 presents data illustrating VMN Ghrh-R gene silencing on VMNdm Ghrh/SF-1 neuron Ghrh-R (Figure 1A), Ghrh (Figure 1C), and SF-1 (Figure 1D) gene expression and Ghrh-R protein (Figure 1B) levels in male or female rats. The outcomes of the statistical analysis of data depicted in Figure 1A are as follows: F_(7,88)_ = 74.77, *p* < 0.001; sex main effect: F_(1,88)_ = 65.28, *p* = 0.001; pretreatment main effect: F_(1,88)_ = 263.92, *p* < 0.001; treatment main effect: F_(1,88)_ = 106.73, *p* < 0.001; sex/pretreatment interaction: F_(1,88)_ = 13.17, *p* < 0.001; sex/treatment interaction: F_(1,88)_ = 62.80, *p* = 0.001; pretreatment/treatment interaction: F_(1,88)_ = 4.42, *p* = 0.04; sex/pretreatment/treatment interaction: F_(1,88)_ = 7.08, *p* = 0.009. The results indicate that for each sex, Ghrh-R siRNA significantly diminished Ghrh-R gene expression in V- [Ghrh-R siRNA/V (purple box-and-whisker plots) versus SCR siRNA/V (green box-and-whisker plots)] or INS- [Ghrh-R siRNA/INS (blue box-and-whisker plots) versus SCR siRNA/INS (brown box-and-whisker plots)]-injected animals. Hypoglycemia up-regulated Ghrh-R mRNA profiles in male [SCR siRNA/INS (brown box-and-whisker plot) versus SCR siRNA/INS (green box-and-whisker plot)], but not female VMNdm Ghrh/SF-1 neurons; Ghrh-R siRNA pretreatment suppressed Ghrh-R transcript levels in hypoglycemic rats of each sex [Ghrh-R siRNA/INS (blue box-and-whisker plot) versus SCR siRNA/INS (brown box-and-whisker plot)]. The data presented in Figure 1B show that VMN Ghrh-R siRNA administration significantly decreased VMNdm Ghrh/SF-1 nerve cell Ghrh-R protein content in eu- and hypoglycemic rats of either sex [F_(7,16)_ = 169.19, *p* < 0.001; sex main effect: F_(1,16)_ = 156.27, *p* < 0.001; pretreatment main effect: F_(1,16)_ = 380.01, *p* < 0.001; treatment main effect: F_(1,16)_ = 228.96, *p* < 0.001; sex/pretreatment interaction: F_(1,16)_ = 46.68, *p* < 0.001; sex/treatment interaction: F_(1,16)_ = 215.82, *p*< 0.001; pretreatment/treatment interaction: F_(1,16)_ = 82.56, *p* < 0.001; sex/pretreatment/treatment interaction: F_(1,16)_ = 74.04, *p* < 0.001]. Hypoglycemia up-regulated this protein profile in male, but not female rats.

The data shown in Figure 1C illustrate VMN Ghrh-R gene-silencing effects on VMNdm Ghrh/SF-1 nerve cell Ghrh mRNA content. These data are statistically analyzed as follows: [F_(7,88)_ = 24.18, *p* < 0.001; sex main effect: F_(1,88)_ = 65.59, *p* < 0.001; pretreatment main effect: F_(1,88)_ = 3.42, *p* = 0.07; treatment main effect: F_(1,88)_ = 1.92, *p* = 0.17; sex/pretreatment interaction: F_(1,88)_ = 63.15, *p* < 0.001; sex/treatment interaction: F_(1,88)_ = 6.04, *p* = 0.016; pretreatment/treatment interaction: F_(1,88)_ = 14.94, *p* < 0.001; sex/pretreatment/treatment interaction: F_(1,88)_ = 14.22, *p* < 0.001. Outcomes indicate that VMN Ghrh-R gene knockdown elevated baseline Ghrh gene expression in males but did not modify this mRNA profile in females. Hypoglycemia did not modify VMNdm Ghrh/SF-1 nerve cell Ghrh transcript levels in either sex, but Ghrh-R siRNA pretreatment had contrary effects on hypoglycemic patterns of Ghrh transcription, namely up- versus down-regulation in INS-injected male versus female rats. Figure 1D depicts effects of intra-VMN Ghrh-R siRNA administration on baseline and hypoglycemic patterns of VMNdm Ghrh/SF-1 neuron SF-1 gene expression in male versus female rats [F_(7,88)_ = 37.69, *p* = <0.001; sex main effect: F_(1,88)_ = 0.002, *p*= 0.962; pretreatment main effect: F_(1,88)_ = 0.11, *p* = 0.742; treatment main effect: F_(1,88)_ = 1.99, *p*= 0.162; sex/pretreatment interaction: F_(1,88)_ = 18.06, *p* < 0.001; sex/treatment interaction: F_(1,88)_ = 10.23, *p* = 0.002; pretreatment/treatment interaction: F_(1,88)_ = 233.41, *p* < 0.002; sex/pretreatment/treatment interaction: F_(1,88)_ = 0.06, *p* = 0.812]. Results show that VMN Ghrh-R gene silencing suppressed SF-1 mRNA levels in V-injected male or female rats and reversed hypoglycemic down-regulation of this gene profile in each sex.

**Figure 2 •:**
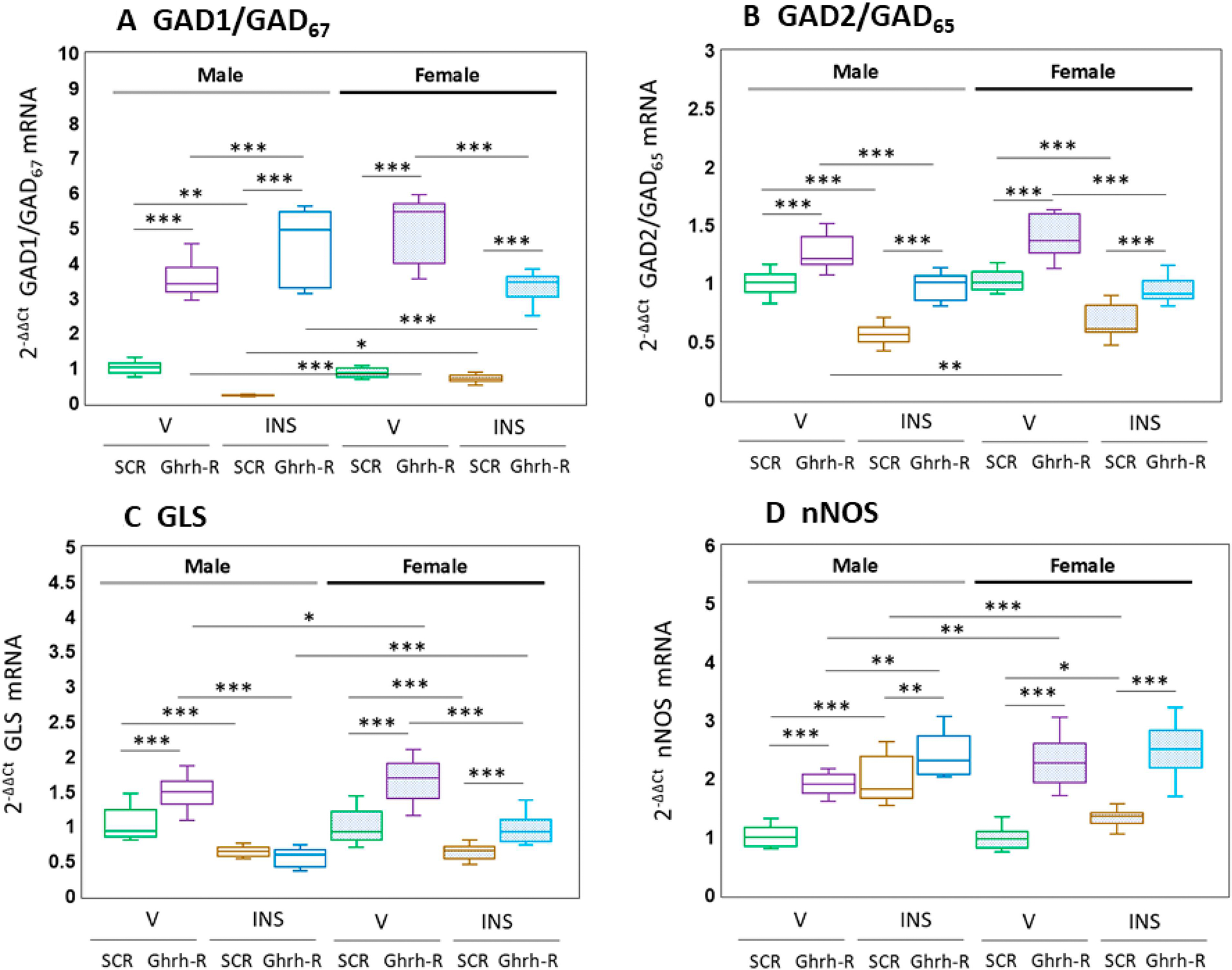
Ghrh-R-dependent VMNdm Ghrh/SF-1 neuron glutamate decarboxylase (GAD)-1/GAD_67_, GAD2/GAD_65_, glutaminase (GLS), and neuronal nitric oxide synthase (nNOS) mRNA profiles in eu- and hypoglycemic male or female rats. The results show normalized GAD1 (**A**), GAD2 (**B**), GLS (**C**), and nNOS (**D**) mRNA values + S.E.M. for male (*at left*) or female (*at right*) groups of rats treated as follows: SCR siRNA/V (green box-and-whisker plots; male: no fill, n = 12; female: stippled fill, n = 12); Ghrh-R siRNA/V (purple box-and-whisker plots; male: no fill, n = 12; female; stipple fill, n = 12); SCR siRNA/INS (brown box-and-whisker plots; male: no fill, n = 12; female: stippled fill, n = 12); Ghrh-R siRNA/INS (blue box-and-whisker plots; male: no fill, n = 12; female: stippled fill, n = 12). Normalized mRNA data were analyzed by three-way ANOVA and Student–Neuman–Keuls post hoc test. Statistical differences between discrete pairs of treatment groups are denoted as follows: * *p* < 0.05; ** *p* < 0.01; *** *p* < 0.001.

VMNdm Ghrh/SF-1 neurons express genes that transcribe protein markers for the counterregulation-constraining or -enhancing amino acid transmitters GABA (GAD1/GAD_67_; GAD2/GAD_65_) and glutamate (GLS), and exhibit transcripts encoding the enzyme nNOS, which produces the gaseous neurotransmitter NO, a positive stimulus for counterregulatory hormone secretion. Figure 2 illustrates VMN Ghrh-R gene knockdown effects on patterns of GAD1/GAD_67_; (Figure 2A), GAD2/GAD_65_ (Figure 2B), glutamate (GLS) (Figure 2C) and nNOS (Figure 2D). Statistical analysis of Figure 2A data are as follows: [F_(7,88)_ = 144.54, *p* < 0.001; Sex main effect: F_(1,88)_ = 2.47, *p* = 0.120; Pretreatment main effect: F_(1,88)_ = 919.83, *p* < 0.001; Treatment main effect: F_(1,88)_ = 15.01, *p* < 0.001; Sex/pretreatment interaction: F_(1,88)_ = 0.01, *p* = 0.916; Sex/treatment interaction: F_(1,88)_ = 20.52, *p* < 0.001; Pretreatment/treatment interaction: F_(1,88)_ = 0.19, *p* = 0.663; Sex/pretreatment/treatment interaction: F_(1,88)_ = 53.77, *p* < 0.001. The results show that hypoglycemia inhibited GAD1 mRNA levels in male, but not female rats. VMN Ghrh-R gene knockdown up-regulated GAD1 gene expression in V- or INS-injected rats of either sex. As shown in Figure 2 [F_(7,88)_ = 35.18, *p* < 0.001; sex main effect: F_(1,88)_ = 3.84, *p* = 0.053; pretreatment main effect: F_(1,88)_ = 101.10, *p* < 0.001; treatment main effect: F_(1,88)_ = 135.80, *p* < 0.001; sex/pretreatment interaction: F_(1,88)_ = 0.12, *p* = 0.725; sex/treatment interaction: F_(1,88)_ = 0.97, *p* = 0.328; pretreatment/treatment interaction: F_(1,88)_ = 0.19, *p* = 0.732; sex/pretreatment/treatment interaction: F_(1,88)_ = 4.29, *p* = 0.041], hypoglycemia down-regulated VMNdm Ghrh/SF-1 neuron GAD2 transcription in each sex. Ghrh-R siRNA enhanced GAD2 gene expression in V- or INS-injected rats of each sex.

The data presented in Figure 2C depict VMN Ghrh-R gene-silencing effects on VMNdm Ghrh/SF-11 nerve cell GLS gene expression [F_(7,88)_ = 48.41, *p* < 0.001; sex main effect: F_(1,88)_ = 10.22, *p* = 0.002; pretreatment main effect: F_(1,88)_ = 71.59, *p* < 0.001; treatment main effect: F_(1,88)_ = 209.31, *p* < 0.001; sex/pretreatment interaction: F_(1,88)_ = 13.94, *p* < 0.001; sex/treatment interaction: F_(1,88)_ = 2.74, *p* = 0.101; pretreatment/treatment interaction: F_(1,88)_ = 29.44, *p* < 0.001; sex/pretreatment/treatment interaction: F_(1,88)_ = 1.63, *p* = 0.205]. The results show that Ghrh-R siRNA increased baseline GLS mRNA transcription in euglycemic male and female rats and attenuated hypoglycemic down-regulation of this gene profile in females, but not males. Figure 2D illustrates VMNdm Ghrh/SF-1 neuron nNOS gene transcription responses to VMN Ghrh-R gene knockdown in each sex [F_(7,88)_ = 46.52, *p* < 0.001; sex main effect: F_(1,88)_ = 0.52, *p* = 0.474; pretreatment main effect: F_(1,88)_ = 220.35, *p* < 0.001; treatment main effect: F_(1,88)_ = 62.80, *p* < 0.001; sex/pretreatment interaction: F_(1,88)_ = 21.97, *p* < 0.001; Sex/treatment interaction: F_(1,88)_ = 12.04, *p* = 0.001; pretreatment/treatment interaction: F_(1,88)_ = 6.31, *p* = 0.014; sex/pretreatment/treatment interaction: F_(1,88)_ = 1.65, *p* = 0.202]. The results indicate that Ghrh-R siRNA increased baseline nNOS gene expression in V-injected animals of either sex and exacerbated hypoglycemic up-regulation of this mRNA profile in male and female rats.

**Figure 3 •:**
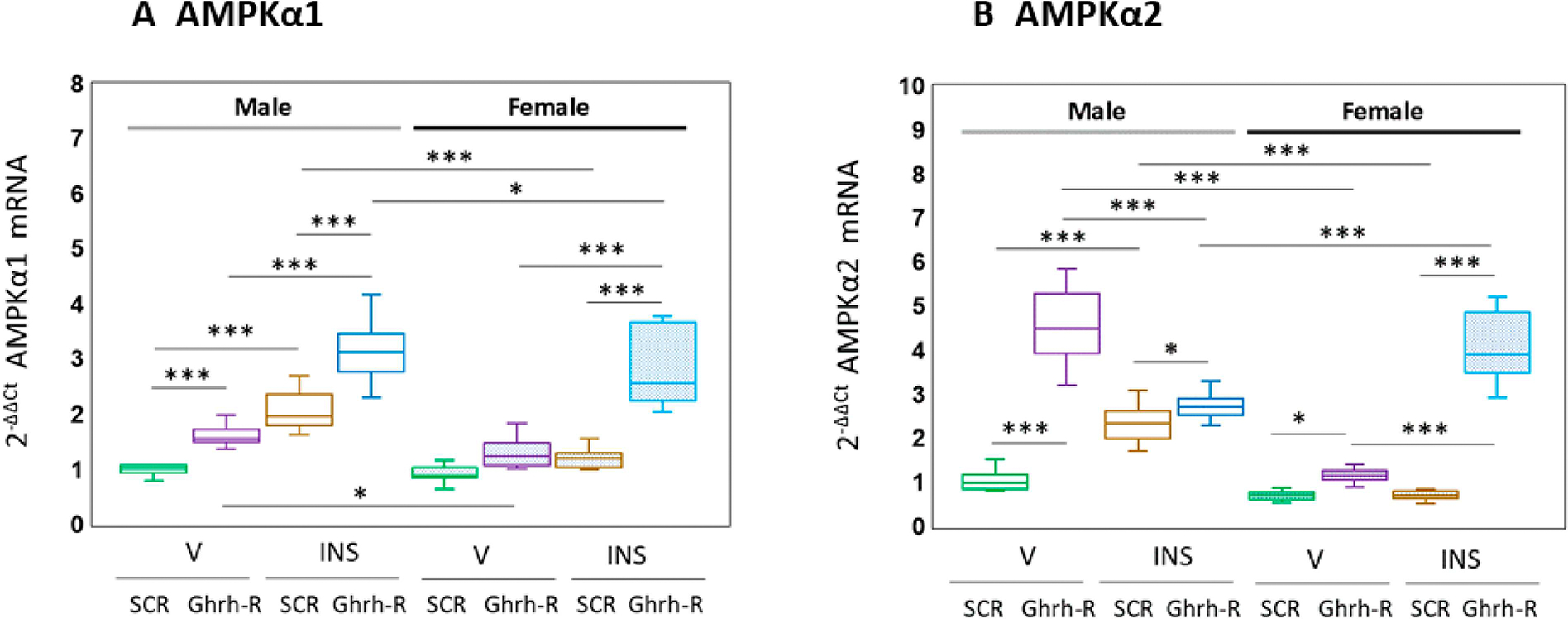
Effects of VMN Ghrh gene knockdown on 5′-AMP-activated protein kinase-alpha1 (AMPKα1) and AMPK-alpha2 (AMPKα2) gene expression in VMNdm Ghrh nerve cells collected from V- or INS-injected male or female rats. Data for normalized Ghrh nerve cell AMPKα1 (3**A**) and AMPKα2 (3**B**) mRNA measures for male (*at left*) or female (*at right*) treatment groups are depicted here in box-and-whisker plot format. Treatment groups are represented as follows: SCR siRNA/V (green box-and-whisker plots; male: no fill, n = 12; female: stippled fill, n = 12); Ghrh-R siRNA/V (purple box-and-whisker plots; male: no fill, n = 12; female; stipple fill, n = 12); SCR siRNA/INS (brown box-and-whisker plots; male: no fill, n = 12; female: stippled fill, n = 12); Ghrh-R siRNA/INS (blue box-and-whisker plots; male: no fill, n = 12; female: stippled fill, n = 12). Normalized mRNA data were analyzed by three-way ANOVA and a Student–Neuman–Keuls post hoc test. Statistical differences between discrete pairs of treatment groups are denoted as follows: * *p* < 0.05; *** *p* < 0.001.

VMNdm Ghrh/SF-1 neurons express the AMPK catalytic subunit variants α1 and −2, which exhibit sex-dimorphic transcriptional responses to Ghrh gene knockdown. The data presented in Figure 3 illustrate the effects of VMN Ghrh-R gene knockdown on expression patterns of these mRNAs in eu- versus hypoglycemic rats of each sex. Statistical analysis of AMPKα1 gene expression data (Figure 3A) produced the following outcomes: [F_(7,88)_ = 64.96, *p* < 0.001; sex main effect: F_(1,88)_ = 31.94, *p* < 0.001; pretreatment main effect: F_(1,88)_ = 154.58, *p* < 0.001; treatment main effect: F_(1,88)_ = 218.52, *p* < 0.001; sex/pretreatment interaction: F_(1,88)_ = 1.08, *p* = 0.302; sex/treatment interaction: F_(1,88)_ = 7.48, *p* = 0.008; pretreatment/treatment interaction: F_(1,88)_ = 34.37, *p* < 0.001; sex/pretreatment/treatment interaction: F_(1,88)_ = 6.75, *p* = 0.011. The results indicate that Ghrh-R gene silencing up-regulated baseline AMPKα1 mRNA levels in V-injected male, but not female rats. Hypoglycemia augmented this gene profile in male rats only; Ghrh-R siRNA pretreatment stimulated AMPKα1 transcription in hypoglycemic animals of each sex. Figure 3B illustrates patterns of VMNdm Ghrh/SF-1 nerve cell AMPKα2 gene expression after SCR versus Ghrh-R siRNA administration to the VMN [F_(7,88)_ = 141.68, *p* < 0.001; sex main effect: F_(1,88)_ = 118.99, *p* < 0.001; pretreatment main effect: F_(1,88)_ = 458.78, *p* < 0.001; treatment main effect: F_(1,88)_ = 46.67, *p* < 0.001; sex/pretreatment interaction: F_(1,88)_ = 0.01, *p* = 0.925; sex/treatment interaction: F_(1,88)_ = 87.47, *p* < 0.001; pretreatment/treatment interaction: F_(1,88)_ = 0.28, *p* = 0.597; sex/pretreatment/treatment interaction: F_(1,88)_ = 279.57, *p* < 0.001. The outcomes indicate that Ghrh-R gene silencing up-regulated baseline AMPKα2 mRNA levels in each sex. Hypoglycemia increased AMPKα2 gene expression in male, but not female rats; Ghrh-R siRNA pretreatment amplified this transcript profile in INS-injected rats of each sex.

**Figure 4 •:**
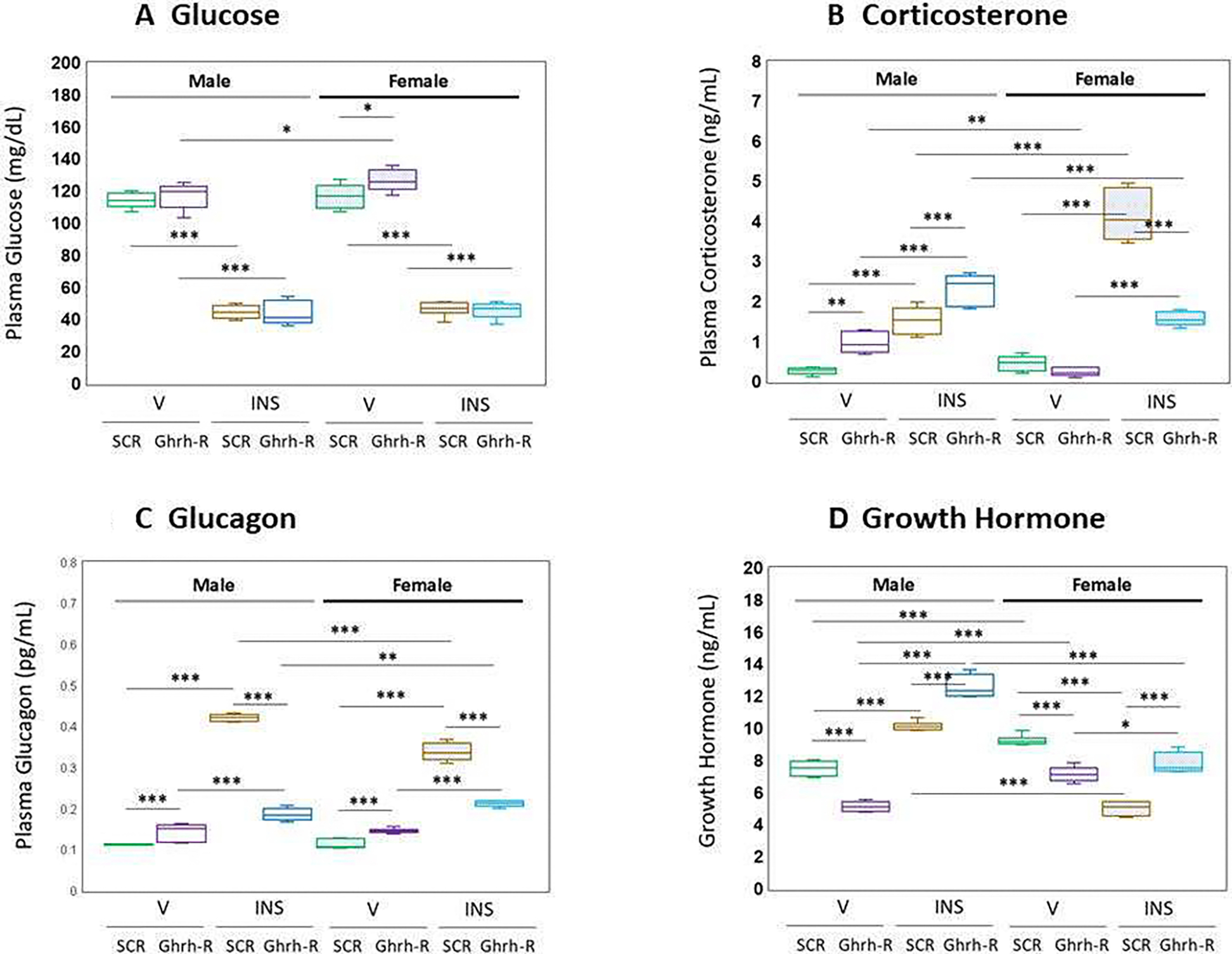
Effects of VMN Ghrh gene knockdown on plasma glucose and counterregulatory hormone profiles in eu- or hypoglycemic male and female rats. Plasma samples were obtained from groups of Ghrh or SCR siRNA-pretreated male and female rats one hour after *sc* injection of V or INS and analyzed for glucose (**A**), corticosterone (**B**), glucagon (**C**), or growth hormone (**D**) concentrations. Treatment groups are represented as follows: SCR siRNA/V (green box-and-whisker plots; male: no fill, n = 6; female: stippled fill, n = 6); Ghrh-R siRNA/V (purple box-and-whisker plots; male: no fill, n = 6; female; stipple fill, n = 6); SCR siRNA/INS (brown box-and-whisker plots; male: no fill, n = 6; female: stippled fill, n = 6); Ghrh-R siRNA/INS (blue box-and-whisker plots; male: no fill, n = 6; female: stippled fill, n = 6). In each panel, individual treatment group data depict mean plasma concentrations + S.E.M. for n = 6 samples. Data were analyzed by three-way ANOVA and Student–Neuman–Keuls post hoc test, using GraphPad Prism, Vol. 8 software. * *p* < 0.05, ** *p* < 0.01, *** *p* < 0.001.

Figure 4 depicts VMN Ghrh-R knockdown effects on circulating glucose (Figure 4A), corticosterone (Figure 4B), glucagon (Figure 4C), and GH (Figure 4D) concentrations. Data in Figure 4A [F_(7,40)_ = 239.00, *p* < 0.001; Sex main effect: F_(1,40)_ = 4.82, *p* = 0.34; Pretreatment main effect: F_(140)_ = 2.26, *p* = 0.141; treatment main effect: F_(1,40)_ = 1558.75, *p* < 0.001; sex/pretreatment interaction: F_(1,40)_ = 0.99, *p* = 0.327; Sex/treatment interaction: F_(1,40)_ = 1.28, *p* = 0.264; Pretreatment/treatment interaction: F_(1,40)_ = 4.04, *p* = 0.051; sex/pretreatment/treatment interaction: F_(1,40)_ = 0.90, *p* = 0.349] show that Ghrh-R gene silencing increased plasma glucose levels in V-injected female, but not male rats. INS administration suppressed plasma glucose concentrations to an equivalent extent in male versus female rats; Ghrh-R siRNA pretreatment did not affect the magnitude of this inhibitory response. As shown in Figure 4B [F_(7,40)_ = 105.04, *p* < 0.001; sex main effect: F_(1,40)_ = 13.12, *p* = 0.001; pretreatment main effect: F F_(1,40)_ = 12.84, *p* = 0.001; treatment main effect: F_(1,40)_ = F_(140)_ = 440.53, *p* < 0.001; sex/pretreatment interaction: F_(1,40)_ = 139.55, *p* < 0.001; sex/treatment interaction: F_(1,40)_ = 44.06, *p* < 0.001; pretreatment/treatment interaction: F_(1,40)_ = 39.11, *p* < 0.001; sex/pretreatment/treatment interaction: F_(1,40)_ = 46.05, *p* < 0.001], plasma corticosterone levels were increased (male) or unaffected (female) in VMN Ghrh-R—pretreated, V-injected rats. Hypoglycemic up-regulation of circulating corticosterone levels was exacerbated (male) or attenuated (female) by Ghrh-R siRNA pretreatment. Data on VMN Ghrh-R gene knockdown effects on plasma glucagon levels are presented in Figure 4C [F_(7,40)_ = 415.17, *p* < 0.001; sex main effect: F_(1,40)_ = 11.77, *p* < 0.001; pretreatment main effect: F_(140)_ = 362.11, *p* < 0.001; treatment main effect: F_(1,40)_ = 1865.88, *p* < 0.001; sex/pretreatment interaction: F_(1,40)_ = 50.48, *p* < 0.001; sex/treatment interaction: F_(1,40)_ = 14.82, *p* < 0.001; pretreatment/treatment interaction: F_(1,40)_ = 733.48, *p* < 0.001; sex/pretreatment/treatment interaction: F_(1,40)_ = 47.61, *p* < 0.001]. Gene silencing elevated basal glucagon secretion in each sex. Hypoglycemic up-regulation of this hormone profile was reversed by Ghrh-R siRNA pretreatment. The data shown in Figure 4D illustrate eu- and hypoglycemic patterns of GH secretion following VMN Ghrh-R gene silencing [F_(7,40)_ = 169.79, *p* < 0.001; sex main effect: F_(1,40)_ = 123.59, *p* < 0.001; pretreatment main effect: F_(140)_ = 2.09, *p* = 0.156; treatment main effect: F_(1,40)_ = 142.56, *p* < 0.001; sex/pretreatment interaction: F_(1,40)_ = 1.28, *p* = 0.265; sex/treatment interaction: F_(1,40)_ = 608.07, *p* < 0.001; pretreatment/treatment interaction: F_(1,40)_ = 310.95, *p* < 0.001; sex/pretreatment/treatment interaction: F_(1,40)_ = 0.01, *p* = 0.913]. The results show that receptor knockdown diminished baseline GH release in each sex. Hypoglycemia correspondingly up- or down-regulated this hormone profile in male versus female rats, respectively. Ghrh-R siRNA pretreatment was observed to enhance GH release in hypoglycemic animals of each sex.

**Figure 5 •:**
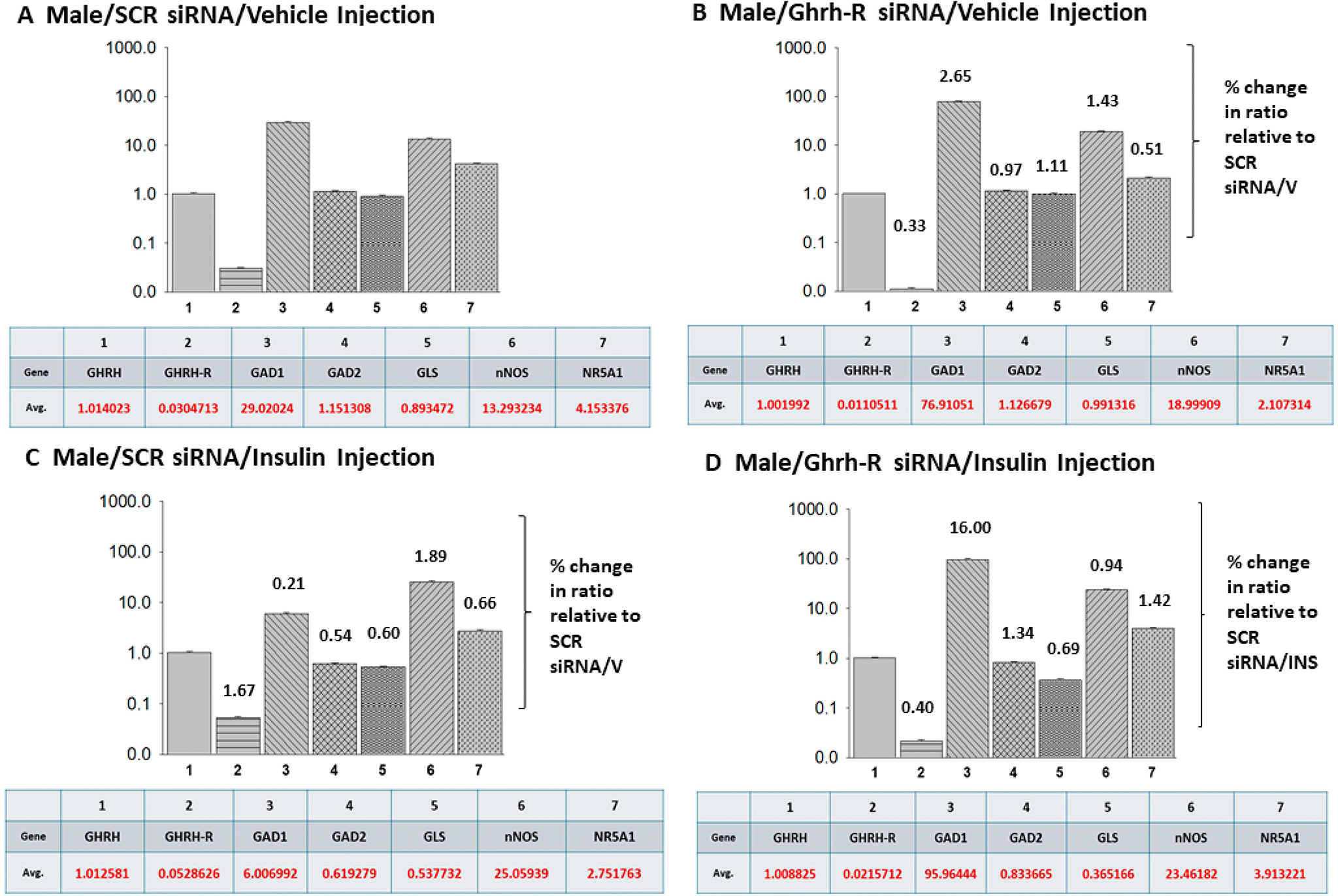
Mean relative expression ratios of counterregulatory transmitter marker, Ghrh-R, and SF-1 mRNAs analyzed for eu- versus hypoglycemic male rat VMNdm Ghrh/SF-1 neurons. Multiplex single-cell qPCR data acquired from laser catapult-microdissected Ghrh–ir neurons were used to establish mean ratios for target gene expression levels relative to Ghrh mRNA. Averaged mean ratio values were derived from n = 12 laser catapult-microdissected VMNdm Ghrh-ir neurons from male rats treated as follows: SCR siRNA/V (**A**), Ghrh-R siRNA/V (**B**), SCR siRNA/INS (**C**), Ghrh-R/INS (**D**). In each figure panel, mean relative gene expression values are depicted in graphical (*top*; bars with S.E.M.; note the exponential Y axis scale) and tabular (*bottom*; (1) top row: gene name; (2) bottom row: average expression ratio value) formats. (**B**,**C**) show a numerical notation above individual relative expression ratio bars to indicate percentage change versus the corresponding target gene/Ghrh mRNA ratio for SCR siRNA/V control group. Notations associated with mean ratio bars in (**D**) indicate the percentage change in proportionate target gene expression compared to corresponding ratios for the SCR siRNA/INS treatment group.

Figure 5 depicts expression ratios of counterregulatory transmitter marker, Ghrh-R, and SF-1/NR5A1 mRNAs in VMNdm Ghrh/SF-1 neurons collected from SCR or Ghrh-R siRNA-pretreated *sc* V- (Figure 5A,B) or INS- (Figure 5C,D)-injected male rats. In each panel, average ratios of individual target gene versus Ghrh mRNA profiles, identified by number, are depicted in graphical (note the exponential Y axis scale) and tabular formats. The data in Figure 5A show that under euglycemic conditions, proportionate target gene expression varies, as indicated by differential average values. Figure 5B depict effects of Ghrh-R knockdown on individual gene expression ratio as well as numerical notation of change in ratio values versus SCR siRNA/V controls. Data show that this treatment exacerbated relative GAD1 and nNOS expression and, at the same time, diminished ratios of Ghrh-R or SF-1 versus Ghrh mRNA. Comparison of VMNdm Ghrh/SF-1 neuron relative target gene expression in hypo- (Figure 5D) versus euglycemic (Figure 5C) animals infers that glucose imbalance augments nNOS/Ghrh and Ghrh-R/Ghrh mRNA ratios, while suppressing proportionate yield of remaining gene transcripts of interest. Interestingly, Ghrh-R siRNA/INS-injected male rats exhibited a gain (GAD2, GLS), switch in direction (SF-1), or loss (nNOS) of control of relative gene expression compared with rats receiving INS treatment in the absence of Ghrh-R gene silencing.

**Figure 6 •:**
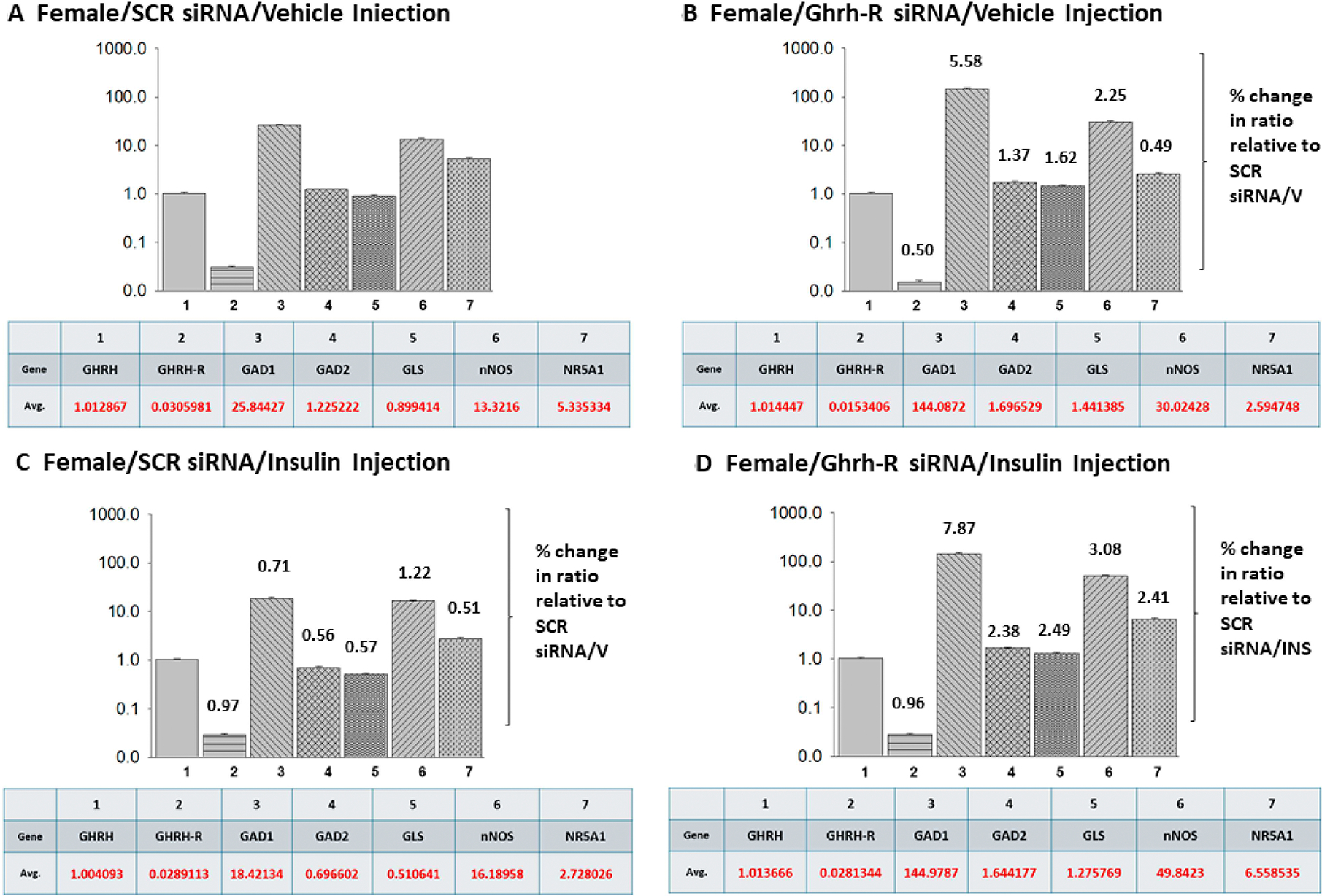
Relative VMNdm Ghrh/SF-1 neuron target gene expression ratios for SCR versus Ghrh-R siRNA-pretreated, V- or INS-injected female rats. Mean ratios for Ghrh/SF-1 nerve cell target gene expression relative to Ghrh mRNA were constructed using multiplex single-cell qPCR data derived from individual laser catapult-microdissected Ghrh–ir neurons harvested from SCR siRNA/V (**A**), Ghrh-R siRNA/V (**B**), SCR siRNA/INS (**C**), or Ghrh-R/INS (**D**) groups of female rats. Mean relative gene expression values for each treatment group are presented in a bar graph (*at the top*) and table (*at the bottom*). (**B**,**C**) show a numerical notation above individual relative expression ratio bars to indicate percentage change versus the corresponding target gene/Ghrh mRNA ratio for the SCR siRNA/V control group. Notations associated with mean ratio bars in (**D**) indicate a percentage change in proportionate target gene expression compared to corresponding ratios for the SCR siRNA/INS treatment group.

The data presented in Figure 6 illustrate target gene versus Ghrh mRNA ratios in VMNdm Ghrh/SF-1 neurons from SCR siRNA/V (Figure 6A), Ghrh-R siRNA/V (Figure 6B), SCR siRNA/INS (Figure 6C) and Ghrh-R siRNA/INS (Figure 6D) female rats. Euglycemic female rats exhibit divergent target gene expression relative to Ghrh (Figure 6A). As in the male, Ghrh-R gene silencing altered the magnitude of each mRNA ratio of interest (Figure 6B), yet the magnitude of adjustment in proportionate Ghrh-R, GAD1, GAD2, GLS, and nNOS gene expression is evidently different from that observed in male rats (Figure 5B). Hypoglycemic female and male rats exhibit similar trends in change in proportionate target gene transcription (Figure 6C compared to Figure 5B), yet the magnitude of reduction in GAD or augmentation of nNOS relative expression varies between the sexes. The data in Figure 6D show that Ghrh-R siRNA pretreatment caused uniform augmentation of proportionate target gene expression, excluding Ghrh-R, in hypoglycemic female rats; notably, these increases were of higher magnitude than those measured in euglycemic, INS-injected animals.

## 4. Discussion

Current research used an in vivo gene knockdown strategy to examine whether Ghrh action within VMN controls counterregulatory hormone and VMNdm Ghrh/SF-1 neuron counterregulatory neurochemical marker gene expression in male and/or female rats. The results document bi-directional, i.e., opposite VMN Ghrh-R regulatory effects on basal versus hypoglycemic patterns of corticosterone (female), glucagon (both sexes) and GH (both sexes) secretion. Single-cell multiplex qPCR analysis of VMNdm Ghrh/SF-1 neurons showed that in each sex, VMN Ghrh-R signaling stimulates (euglycemia) or inhibits (hypoglycemia) SF-1 gene expression. This receptor imposes a uniform inhibitory tone on GAD1/GAD_67_, GAD2/GAD_65_, and nNOS mRNAs in male and female rats regardless of plasma glucose profiles, but systemic glucose status affects Ghrh-R regulation of Ghrh (female) and GLS (male) transcription in these neurons. Ghrh-R regulation of AMPKα1, but not AMPKα2 mRNA levels varies between eu- versus hypoglycemia female rats. Study outcomes uniquely document glucose-dependent VMN Ghrh-R control of counterregulatory hormone secretion and distinctive VMNdm neuron counterregulatory transmitter marker gene profiles in each sex. Present data support the prospect that this receptor may shape VMNdm Ghrh neuron adaptation to glucose dyshomeostasis by SF-1 (both sexes)- and AMPKα1 (females)-dependent mechanisms.

Present studies are notable for affirming that VMNdm Ghrh/SF-1 neurons are directly receptive to Ghrh and advancing the novel concept that Ghrh-R regulation of the VMN-specific metabolic nuclear transcription factor SF-1, energy sensor, and counterregulatory neurotransmitter functions in this neuron population is controlled by systemic glucose status. Moreover, outcomes are notable for documenting sex-dimorphic aspects of this control. The results reported here underscore the need to determine how IIH-associated decrements in plasma glycemic profiles may affect VMN Ghrh-R signaling in each sex. Hypoglycemia was found to cause sex-specific changes in VMNdm Ghrh/SF-1 neuron Ghrh-R mRNA levels, as these transcripts were up-regulated in males but were unaffected by this metabolic perturbance in females. Thus, in males, hypoglycemia-associated enhancement of cellular sensitivity and associated receptor signal volume may contribute to differential Ghrh-R-dependent neurochemical marker gene expression during eu- versus hypoglycemia. There is a justifiable need to characterize the post-receptor signaling mechanism(s) that function in each sex to link Ghrh-R to gene transcription regulation. VMNdm Ghrh/SF-1 neurons express transcripts that encode hypoglycemia-sensitive metabolic sensory biomarkers, namely the glucose sensor glucokinase and energy sensor AMPK alpha subunit isoforms [35]. It would be informative to determine if hypoglycemia-associated changes in VMNdm Ghrh/SF-1 neuron uptake and metabolism of glucose and/or nerve cell energy stability may affect the volume or direction of activity of Ghrh-R-sensitive signal transduction pathways according to sex. It is reasonable to presume that Ghrh-R regulation of VMNdm Ghrh/SF-1 nerve cell gene transcription is mediated, in part, by direct neuropeptide action on those cells. However, the possibility that Ghrh may control gene expression in Ghrh/SF-1 neurons via afferent input from other Ghrh-R-expressing VMN neurons, under eu and/or hypoglycemic conditions, should not be overlooked.

VMN Ghrh-R gene silencing had variable effects on baseline VMNdm Ghrh/SF-1 neuron Ghrh gene expression in male (diminished) versus female (unchanged) rats, inferring that this receptor may impose a tonic inhibitory feedback tone on Ghrh neurotransmission by these cells in one, but not both sexes. The plausible supposition that Ghrh neuropeptide expression is correspondingly inhibited by Ghrh-R signaling in euglycemic male, but not female rats will require experimental confirmation by application of analytical methods of requisite sensitivity for protein quantification in single brain cell samples. These sex-dependent Ghrh mRNA responses to equivalent reductions in VMNdm Ghrh/SF-1 neuron Ghrh-R gene expression may likely reflect, in part, dissimilar post-receptor signaling responses to diminished Ghrh-R receptor activity. In addition to the above-noted need for research to discern the signal transduction mechanism(s) that mediate Ghrh-R effects on this neuron population, it will also be important to clarify how that/those pathway(s) may be subject to sex-specific control in the presence of normoglycemia. The results also disclose opposite Ghrh-R regulatory effects on Ghrh gene expression in hypoglycemic male (inhibitory tone) versus female (stimulatory tone) rats. Female VMNdm Ghrh/SF-1 neurons exhibit an apparent gain in positive Ghrh-R regulatory tone during hypoglycemia. Thus, in this sex, it will be imperative to learn how glucose sufficiency versus deficiency affects Ghrh-R control of Ghrh gene transcription. As net Ghrh protein levels were not different between V-versus INS-injected animals in either sex, the suppressive (male) or augmenting (female) effects of Ghrh-R input are evidently offset by other stimuli that regulate this neuropeptide profile.

Co-transmission of counterregulation-inhibiting and -stimulating neurochemicals of diverse chemical structure, spatial, and temporal profiles likely allows VMNdm Ghrh/SF-1 neurons to deliver complex, dynamic sex-conditional input to the brain’s glucostatic circuitry. It would be informative to learn if individual transmitters released by these cells convey distinguishing or redundant information on dynamic aspects of brain cell energy state, metabolic fuel supply, and local/systemic energy reserve capacities. There is a consensus that the amino acid transmitter GABA acts to inhibit counterregulatory hormone secretion. The data acquired here show that in both sexes, VMN Ghrh-R enforces a uniform negative regulatory tone on GAD1/GAD_67_ and GAD2/ GAD_65_ gene expression, irrespective of plasma glucose levels. These findings support the view that this receptor may function, in part, to suppress tonic VMNdm Ghrh/SF-1 nerve cell GABAergic neurotransmission, and moreover, to diminish this neurochemical signal below baseline during hypoglycemia. This supposition requires experimental verification that down-regulated Ghrh-R gene expression elicits concurrent reductions in GAD variant protein and GABA production of comparable magnitude. Existing studies do not shed light on where Ghrh-R-controlled VMNdm Ghrh nerve cell GABA release may occur within the brain glucose-regulatory circuitry. Expression of mRNA encoding GLS, a protein marker for the counterregulatory-enhancing amino acid glutamate, is also evidently subject to tonic inhibition by Ghrh-R. Current data suggest that hypoglycemic suppression of GLS mRNA in each sex apparently involves Ghrh-R-dependent or -independent mechanisms in female or male rats, respectively. The labile gas NO has documented stimulatory action on counterregulatory hormone secretion.

The present data show that VMN Ghrh-R gene knockdown uniformly augments VMNdm Ghrh/SF-1 neuron nNOS gene expression in both sexes, regardless of systemic glucose status. These findings infer that this receptor may suppress baseline NO production and blunt hypoglycemia-associated up-regulated NO release. Current work does not shed light on the regulatory factor(s) that increase nNOS mRNA profiles in response to lowered plasma glucose concentrations. There remains a need to identify cellular targets and functional sequelae of individual Ghrh-R-controlled Ghrh/SF-1 neuron counterregulatory neurochemical signaling, including investigation of whether one or more glucose-dependent neurotransmitters may mediate Ghrh-R control of counterregulatory hormone release. The data in Figure 5 and Figure 6 depict mean relative expression of counterregulatory marker genes in VMNdm Ghrh/SF-1 neurons harvested from treatment groups composed of male versus female rats, respectively. In each sex, target genes exhibit divergent baseline expression profiles relative to Ghrh mRNA, findings that have potential implications for net integrated input to the brain glucostatic regulatory circuitry. Hypoglycemia-associated adjustments in the magnitude of individual counterregulatory marker gene expression ratios will presumably affect such input. The outcomes here bolster the role of glucose status as a determinant of Ghrh/SF-1 signaling as Ghrh-R regulation of proportionate target gene expression is evidently variable between eu- versus hypoglycemia, albeit in a sex-specific manner.

Current data show that VMN Ghrh-R up-regulates VMNdm Ghrh/SF-1 neuron SF-1 gene expression in each sex when circulating glucose levels occur within the normal range, but this receptor paradoxically inhibits this gene profile in hypoglycemic rats of each sex. Ongoing work aims to characterize the probable molecular mechanism(s) that govern this switch from positive-to-negative regulatory tone. As discussed above, present findings should be extended by efforts to determine if Ghrh-R elicits parallel adjustments in SF-1 gene and protein expression in individual VMNdm Ghrh/SF-1 neurons. The prospect that Ghrh-R may regulate this gene profile, in part, by indirect, i.e., afferent stimulation by other Ghrh-sensitive VMN neurons should be considered. Recent work documents SF-1 control of VMNdm Ghrh/SF-1 nerve cell counterregulatory transmitter marker and AMPK alpha subunit mRNA profiles [35]. Our research did not address the intriguing prospect that Ghrh-R-controlled SF-1 transcriptional activity may shape, albeit in a sex-contingent manner, Ghrh-R-dependent neurotransmitter marker or AMPK gene expression patterns during eu- versus hypoglycemia. There remains a need for future work to investigate if and how downstream molecular effectors of SF-1 may be affected by experimental manipulation of Ghrh-R signaling in Ghrh/SF-1 neurons

AMPKα1 and AMPKα2 catalytic subunits of the ultra-sensitive energy monitor AMPK are activated to a comparable extent in response to elevated intracellular AMP levels, but their dissimilar substrate specificity likely results in differential effects on cell function [34]. Our data indicate that VMN Ghrh-R signaling represses baseline VMNdm Ghrh/SF-1 nerve cell AMPKα1 mRNA profiles in male, but not female rats, but attenuates (male) or prevents (female) hypoglycemia up-regulation of this AMPK alpha subunit. Thus, in the female, Ghrh-R regulation of AMPKα1 gene expression is apparently glucose-dependent, as hypoglycemia is associated with a gain in Ghrh-R negative control. Notably, this inhibitory receptor influence is sufficient to avert changes in expression relative to euglycemic controls, which infers that it imposes primary control of this gene profile during glucose deficiency. In contrast, both sexes exhibit Ghrh-R-mediated suppression of basal AMPKα2 gene expression patterns. While this negative receptor control of AMPKα2 mRNA persists in the hypoglycemic female, effectively preventing transcriptional reactivity to hypoglycemia, it is lost in hypoglycemic male rats.

Evidence for up-regulated AMPK alpha variant gene expression in males, but not females infers that net energy sensor activation may be comparatively greater in male versus female VMNdm Ghrh/SF-1 neurons. This interpretation will necessitate additional experimentation to affirm that AMPKα1 and AMPKα2 total and phosphorylated protein profiles are likewise affected by hypoglycemia in a sex-contingent manner. Likewise, the probability that the degree of hypoglycemia-associated energy imbalance in VMNdm Ghrh/SF-1 neurons differs between the two sexes will require application of as-yet-unavailable analytical tools of requisite sensitivity for quantification of AMP and ATP molecules in single nerve cell samples. It is intriguing, moreover, to consider whether Ghrh-R-mediated normalization of AMPKα1 and AMPKα2 mRNA expression in hypoglycemic females may involve receptor-initiated mechanisms that stabilize energy balance in that sex. Further research is required to elucidate the molecular mechanisms, including potential signal transduction pathways that transduce effects of Ghrh-R activation on AMPKα1 and AMPKα2 gene expression. Documentation of Ghrh-R control of AMPK catalytic subunit gene transcription supports the prospect that AMPK activity controlled by one or both protein variants may mediate receptor effects on Ghrh/SF-1 neuron transmission patterns. Yet, it should be noted that definitive evidence to support that premise will require additional research involving determination of Ghrh-R gene knockdown effects on downstream mediators of AMPK activity in this nerve cell population.

Our research offers unique documentation of VMN Ghrh-R regulation of in vivo counterregulatory hormone secretion patterns in each sex. The data presented here show that receptor gene knockdown failed to affect eu- or hypoglycemic profiles, yet in females, basal or hypoglycemia-associated glucose levels were up-regulated or refractory to Ghrh-R siRNA. These data infer that VMN Ghrh-R stimulation may serve to prevent hyperglycemia in females only, whereas this break is negligible when glucose levels are low. Interestingly, Ghrh-R was found to impose a negative regulatory tone on corticosterone secretion in eu- and hypoglycemic male rats, yet the females exhibited glucose-dependent receptor control involving a gain of positive impact on this hormone profile during hypoglycemia. Notably, this receptor has opposite actions on hypoglycemic hypercorticosteronemia in the two sexes, as it limits the extent of this hormonal response in the male while conversely stimulating corticosterone secretion in females. In each sex, plasma glucagon reactivity to VMN Ghrh-R gene silencing differed when glucose levels were in versus below the normal range. Ghrh-R was observed to inhibit baseline glucagon secretion, yet it up-regulated hormone release during hypoglycemia. VMN Ghrh-R regulation of GH secretion is also glucose-dependent, as this receptor correspondingly increases or decreases hormone output in eu- versus hypoglycemic rats. Present data confirm prior reports that hypoglycemia elicits contrary, sex-specific changes in GH secretion, as this hormone profile was correspondingly up- (males) or down- (females) regulated after INS injection [16]. Thus, Ghrh-R blunts the rise in plasma GH in hypoglycemic males yet causes this hormone profile to decline in females. An unresolved question that merits consideration is whether VMN Ghrh-R that control basal and/or hypoglycemic counterregulatory secretion patterns are expressed only by VMNdm Ghrh/SF-1 neurons, or if Ghrh action on other VMN neuron populations contributes to documented receptor regulation of these hormone profiles. Our research did not address the prominent issue of if and how Ghrh-R signaling may affect the time course of restoration of normoglycemia after insulin injection; such information should be clearly relevant in a clinical setting.

The several examples of sex differences in target gene responses to VMN Ghrh-R gene silencing described here raise the interesting prospect that sex steroid hormones and their associated receptors may mediate discrepant transcriptional reactivity in the two sexes. VMN Ghrh/SF-1 neurons are directly responsive to estradiol due to expression of genes that encode nuclear (ERα and ERβ) and membrane (G protein-coupled estrogen receptor-1) estrogen receptors (ERs) [16]. Brain ERs are stimulated by a ligand that is taken up from the systemic circulation or is produced in local tissue. In the brain, the androgen hormone testosterone is metabolized by aromatase enzyme-catalyzed action to neuroestradiol. Aromatase protein content and enzyme activity profiles are heterogeneous across brain structures, with highest tissue protein levels reported for a set of forebrain loci, namely the medial preoptic area, bed nucleus of the stria terminalis, medial amygdala, and VMN. Recent studies provide evidence to support a role for VMN neuroestradiol in neural governance of glucose counterregulation. Clearly, additional research is needed to examine whether VMNdm Ghrh/SF-1 neurons express aromatase mRNA and whether this transcript profile may be subject to glucose-dependent Ghrh-R control according to sex.

In summary, current studies provide novel evidence that VMN Ghrh-R imposes glucose status control on secretion of the critical counterregulatory hormones corticosterone, glucagon, and GH in one or both sexes. Prior research disclosed that VMNdm Ghrh/SF-1 neurons express Ghrh-R. The evidence acquired here shows that Ghrh-R regulation of genes encoding the VMN-specific metabolic nuclear transcription factor SF-1, distinctive counterregulatory neurotransmitters, or the AMPK catalytic subunit α1 variant is determined by plasma glucose profiles in one or both sexes. There is an evident need for future research to identify the signal transduction pathway(s) that link Ghrh-R activity to VMNdm Ghrh/SF-1 neuron gene transcription regulation, and, furthermore, to elucidate the mechanisms in each sex that govern baseline post-receptor signaling and shape pathway adaptation to hypoglycemia.

## Figures and Tables

**Table 1 •:** Experimental design.

Sc injection; day 7	siRNA pretreatment; day 1		
		SCR siRNA ^a^	Ghrh-R siRNA ^b^
	Vehicle (V) ^c^	Male SCR/V; n = 6 Female SCR/V; n = 6	Male Ghrh-R/V; n = 6 Female Ghrh-R/V; n = 6
	Insulin (INS) ^d^	Male SCR/INS; n = 6 Female SCR/INS; n = 6	Male Ghrh-R/INS; n = 6 Female Ghrh-R/INS; n = 6

a 500 pmol; Accell Control Pool Non-Targeting; prod. no. D-001910-10-20 (Horizon Discovery);

b 500 pmol; Accell siRNA Rat Ghrh-receptor (Ghrh-R), set of 4; prod. no. EQ-099803-00-0010 (Horizon Discovery);

c sterile diluent; 100 uL/100 g bw;

d 10.0 U neutral protamine Hagedorn insulin/kg bw.

## Data Availability

The data that support the findings of this study are available from the corresponding author upon reasonable request.

## Abbreviations

GAD1/GAD_67_: glutamine decarboxylase (GAD)-1/GAD_67_
GAD2/GAD_65_: GAD2/GAD_65_
GH: Growth hormone
Ghrh: Growth hormone-releasing hormone
Ghrh-R: Ghrh receptor
GLS: Glutaminase
IIH: Insulin-induced hypoglycemia
INS: Insulin
nNOS: Neuronal nitric oxide synthase
NO: Nitric oxide
OVX: Ovariectomy
sc: Subcutaneous
SF-1/Nr5a1: steroidogenic factor-1
